# *Weissella confusa*: problems with identification of an opportunistic pathogen that has been found in fermented foods and proposed as a probiotic

**DOI:** 10.3389/fmicb.2014.00254

**Published:** 2014-06-12

**Authors:** Marilynn R. Fairfax, Paul R. Lephart, Hossein Salimnia

**Affiliations:** ^1^Department of Pathology, Wayne State University School of MedicineDetroit, MI, USA; ^2^Clinical Microbiology Division, Detroit Medical Center University LaboratoriesDetroit, MI, USA

**Keywords:** *Weissella confusa* bacteremia, *Weissella confusa* identification, MALDI-TOF mass spectrometry, 16S rDNA sequence, *Weissella confusa* susceptibiliy testing

## Abstract

*Weissella confusa* is found in fermented foods and has been suggested as a probiotic, but also causes sepsis and other serious infections in humans and animals. The incidence of human infections is underestimated partly due to confusion with viridans streptococci and partly due to difficulty making a definitive identification, even if the organism is recognized to belong to another genus, owing to the inability of commercial organism systems to identify it. We report our experiences identifying *W. confusa* isolated from two immune-compromised patients, both of whom developed sepsis with this organism. Two MicroScan gram positive combination panels, could not identify the organism because they did not have *W. confusa* in their data bases, but did not provide a false identification. Other laboratorians have reported failure to identify or false identifications of *W. confusa* with other commercial systems. *W. confusa* is in the data base of the RapID™ Str panel (Remel), which gave three incorrect, high probability results (≥95%). 16S rDNA sequencing identified the isolates as *W. confusa*. Maldi-Tof, performed by two of our reference laboratories, also correctly identified both isolates. Use of *W. confusa* as a probiotic should be approached with caution because its true incidence as an opportunisitic pathogen is unknown.

## Introduction

A search in the PubMed data base for “*Weissella confusa*,” performed on 24 June, 2013, revealed 74 articles about this organism. Forty of them highlighted the finding of *W. confusa* in fermented food, not including the initial paper by Collins et al. (1993) who constructed the genus *Weissella* to include some organisms found in fermented food sources. Others discussed its potential as a probiotic (for example: Aveni et al., 2001; Lee et al., 2012). Two species have been associated with human bacteremia: *W. confusa* (formerly *Lactobacillus confusus*) and *W. cibaria*. *W. confusa* infection is rare which may be due, in part, to the inability of commercial systems to identify the organism. However there were 10 papers highlighting its role as a cause of human sepsis and bacteremia (Green et al., 1990; Olano et al., 2001; Bjorkroth et al., 2002; Flaherty et al., 2007; Salimnia et al., 2010; Shin et al., 2007; Harlan et al., 2011; Kumar et al., 2011; Lee et al., 2011; Fairfax and Salimnia, 2012). These are mostly individual case reports from immune-compromised hosts. However, one paper from Taiwan reports a series of 10 bacteremias over a 10-year period (Lee et al., 2011). About half of these were mixed infections in which the role of the *W. confusa* was undeterminable. Many of the patients died, but whether the outcome could be attributed directly to the *W. confusa* is also uncertain.

We discussed our two *W. confusa* isolates from septic patients in two previous papers. The first highlights the isolation of the organism from two immune-compromised patients (Salimnia et al., 2010). The second (Fairfax and Salimnia, 2012) emphasizes that the organism is probably under-reported because, by spot testing, it resembles a viridans streptococcus (Ruoff, 2002) and therefore may be reported as a skin contaminant and not worked up (Hall and Lyman, 2006). However, this is not the only problem with the identification of *W. confusa*. Many commercial organism identification systems fail to identify or mis-identify the organism (Salimnia et al., 2010; Lee et al., 2011; Fairfax and Salimnia, 2012). For an organism that is a candidate for inclusion in probiotic regimens, this may be dangerous, as its true pathogenic potential may not be appreciated. Our objectives with this paper are to highlight our difficulties with the organism identification in a clinical laboratory, to describe their possible resolutions, and to emphasize the importance of delineating the role of this opportunistic pathogen before deciding that it might be used as a probiotic.

## Case reports

### Patient 1

A 34-year-old man received a successful allogeneic, unrelated hematopoetic stem cell transplant for acute lymphocytic leukemia and was discharged from the hospital. On day 23 post transplant, he returned with fever, neutropenia, and graft failure. One blood culture set was drawn. Empiric therapy with vancomycin (VAN) and aztreonam was begun. Both the aerobic and anaerobic bottles grew gram-positive cocci/coccobacilli in pairs and chains. Although initially presumed to be a viridans streptococcus because of its alpha-hemolytic colonies and spot testing results (Ruoff, 2002) and a blood culture contaminant because it grew in only one culture set (Hall and Lyman, 2006), the organism was worked up at physician request. Two additional blood cultures, drawn when the first set turned positive, grew the same organism. The MicroScan (MS; Siemens Healthcare Diagnostics, Sacramento, CA, USA) PC20 panel showed the organism to be resistant (R) to VAN, metronidazole, and rifampin, and susceptible (S) to other antibiotics on the panel appropriate for the treatment of gram positive cocci. However, it gave no identification. Etest (AB Biodisk, Solna SW) confirmed the VAN resistance and revealed that the organism was S to daptomycin (DAP). DAP therapy was begun and follow up cultures were negative.

### Patient 2

A 58-year-old man with 2nd and 3rd degree burns received extensive debridement, multiple skin grafts, a tracheotomy and prolonged mechanical ventilation. Several bouts of polymicrobial bacteremia were treated with multiple courses of antibiotics including VAN. On hospital day 29, he again developed bacteremia with both *E. faecalis* and a catalase-negative, gram-positive coccobacillus that grew as pairs and chains both aerobically and anaerobically. Identification and susceptibility testing were similar to those for Patient 1. His bacteremia resolved after treatment with DAP.

## Materials and methods

### Blood cultures

All blood culture bottles were incubated in the Bactec 9240 blood culture system (Becton Dickinson, Sparks, MD, USA) according to the manufacturer's instructions.

### Spot tests

The following spot tests were performed according to the manfacturers' instructions: leucine amino peptidase (LAP Disk, Remel, Lenexa, KS, USA), pyrrolidinyl aralamidase (PYR, Identicult®-AE-PYR; PML Microbiologicals, Tualitin OR, USA).

### Commercial identification and susceptibility testing

The first organisms was tested on the MS PC20 panel. The second organism was inoculated into the MS PC29 Panel to which our laboratory had switched in the interim. After the MicroScan failed to identify the organisms, we used the RapID™ Str (RapID, Remel, Lenexa, Kansas, USA), according to the manufacturers' instructions, to identify the organism. *W. confusa* is in the data base of the panel.

*E*-tests for VAN and DAP (A B Biodisc, Solna, SW) were performed according to the manufacturers instructions. The VAN and DAP minimal inhibitory concentrations were ≥256 μgm ml^−1^ and 0.5 μgm ml^−1^ respectively.

### 16S rDNA sequencing

16S rDNA sequencing was performed using the MicroSeq Full Gene 16S rDNA Bacterial Identification Kit (Applied Biosystems). BLAST analysis was used to identify the organism (Nucleotide collection (nr/nt) database, at the NCBI website; http://blast.ncbi.nlm.nih.gov/Blast.cgi).

## Results

The MS gram-positive panels gave antibiotic susceptibility results but failed to identify the organism. Several attempts at organism identification using RapID gave three incorrect identifications depending on the interpretation of ambiguous reactions, all at probabilities greater than 95%: *Streptococcus intermedius, Listeria monocytogenes, Pediococcus pentosaceus* (Table 1). On RapID, *W. confusa* should have been positive for arginine (ARG) and esculin (ESC) hydrolysis, and given negative reactions for all other biochemicals (Table 1). The panel was run twice, on isolates from different days, by different experienced technologists. The first time the ARG, ESC, p-nitro-phenyl-α,D-glucoside (GLU), -glucosaminide (NAG) and p-nitrophenyl phosphate (PO4) were positive. This yielded an identification of *Streptococcus intermedius* with a probability of 95.8%, although the web site cautioned that lysine-negative isolates were uncommon. The second time, ARG and PO4 hydrolysis were negative and GLU and NAG were ambiguous. If GLU and NAG were interpreted as positive, the isolate should have been *Listeria monocytogen*es. If both were negative, it would have coded as *Pediococcus pentosaceus*. These organisms were eliminated by colony and/or gram stain morphology, and by the results from the MS panels. The organism was subjected to rDNA sequencing.

**Table 1:** **Biochemical reactions of *Weissella confusa* isolates from Patients 1 and 2 compared with identification criteria from commercial identification systems**.

**Testing modality**	**Reaction**	**Pt 1**	**Pt 2**	**RapID™ STR**	**MS**	**MS**	***P. pentosaceus***
		Wc^*^	**Wc**	Wc^**^	**E. gal^3^**	**P. sp^4^**	**Koneman^Ô^**
Blood agar	Hemolysis	α	α		α, γ	α	
Spot tests	Catalase	−	−	−	−	−	−
	LAP^6^	+	+				+
	PYR^7^	−	−				
	Gram stain^8^	+c/cbpr and ch	+c/cbpr and ch		+cpr and ch	+cclus	+cclus
Microscan PC20 and PC29^9^	BE	+	+		95	10	
	NACL	+	+		99	1	
	PYR^10^	−	−		75	1	−
	NOV	+	+		10	25	+
	ARG	+	+		50	5	+
	BAC	+	+		99	95	
	CV	−	−		95	1	
	OPT	+	+		99	95	
	MS	+	+		99	90	
	NIT	−	−		1	1	
	PGR	−	−		5	1	
	IDX	−	−		10	1	
	VP	−	+		95	1	
	PHO	−	−		25	1	
	PGT	+	+		99	90	
	URE	−	−		1	1	
	MAN^10^	−	−		99	5	
	LAC	−	−		99	5	
	TRE	−	−		99	90	
	MNS	+	+		99	99	
	SOR^10^	−	−		1	5	
	ARA	−	−		90	90	
	RBS	−	−		5	95	
	INU^10^	−	−		10	1	
	RAF^10^	−	−		50	1	
	PRV	−	−		1	1	RapID™ Str
RapID™ Str	ARG^11^	±^12^	+	95			2
	ESC	+	+	98			96
	RAF	−	−	0			0
	GAL	−	+	0			0
	GLU	+/±^13^	−	0			0
	NAG	+/±	−	2			2
	PO4	−	±	0			0
	TYR	−	−	1			1
	HPR	−	−	0			0
	LYS	−	−	0			0

* Wc: Weissella confusa.

** W. confusa biochemical reactions according to the package insert with the RapID™ STR panel.

3 E. gal: Enterococcus gallinarum biochemical reactions as shown in MicroScan literature.

4 Pediococcus species biochemical reactions as shown in MicroScan literature.

Ô P. pentosaceus biochemical reactions from Winn et al. (2006).

6 LAP, leucine aminopeptidase.

7 PYR, pyrrolidinyl arylamidase.

8 Abbreviations for the gram stain interpretation are: c, cocci; cb, coccobacilli; pr, pairs; ch, chains; clus, clusters.

9 The reagent names on the MicroScan panels are as follows: BE, 40% bile esculin; NACL, 6.5% sodium chloride; PYR, L-pyrrolidonyl-β-naphthylamide; NOV, novobiocin, ARG, arginine; BAC, bacitracin; CV, crystal violet; OPT, optochin; MS, Micrococcus screen, NIT, nitrate; PGR, PNP-β-D-glucuronide; IDX, indoxyl phosphatase; VP, Voges-Proskauer; PGT, PNP-β-D-galactopyranoside, URE, urea; MAN, mannitol; LAC, lactose; TRE, trehalose; MNS, mannose; SOR, sorbitol; ARA, arabinose, RBS, ribose; INU, Inulin; PRV, pyruvate. All the biochemical reagents from the Microscan PC20 and 29 panels are included here, but they have been reordered to place those that we consider most important for Weissella confusa identification at the top of the column.

10 These reagents appear both in the Microscan and RapID™ Str panels, were concordant and negative for both isolates, and were supposed to give a negative reaction for W. confusa according to the RapID™ Str panel information. Accordingly they were deleted from RapID™ Str reagent list to avoid duplication.

11 Reagent names from the RapID™ Str panel are as follows: ARG, L arginine; ESC, esculin; RAF, raffinose; GAL, p-nitrophenyl-α,D-galactoside; GLU, p-nitrophenyl-α,D-glucoside; PO_4_, p-nitrophenyl phosphate; TYR, tyrosine β-naphthylamide; HPR, hydroxyproline β-naphthylamide; LYS, lysine β-naphthylamide. See also footnote 10.

12 Different reactions from two different blood culture isolates from patient one, drawn 2 days apart.

13 ± Ambiguous reaction requiring subjective interpretation.

The sequence obtained by 16S rDNA sequencing had 100% identity to *W. confusa* strain Inje LM S-338 (DQ321751.1) with no gaps and a maximum score of 2754.

The organism from the second patient was similar in colony and gram stain morphology and in spot test results to the isolate from patient 1. However it was not assumed to be a contaminant as it grew in both bottles of two different blood cultures. It was analyzed on the Microscan MS PC29 panel which also did not identify the isolate. The biochemical and antibiotic susceptibility results were similar to those of the first isolate, except for a positive Voges-Proskauer (VP, acetoin) reaction. The *W. confusa* isolate reported by Olano et al. (2001), and those in references cited by them, gave negative VP reactions. It was also sent for 16S rDNA analysis and the sequence was 100% identical to the first patient's isolate. It was then inoculated into the RapID panel, which again failed to support an identification as *W. confusa* (Table 1).

## Discussion

*Weissella confusa* is appropriately named. Difficulty identifying it by standard clinical laboratory techniques may contribute to an underappreciation of its role as an opportunistic pathogen. This is highlighted by our experience with two cases of *W. confusa* causing serious infections in immune compromised patients. Initially thought to be viridans streptococci, and skin contaminants because they were catalase and PYR negative and LAP positive (Ruoff, 2002), the isolates from our two immune-compromised patients might have been dismissed as contaminants if the physician for the first had not requested identification and if the second had not grown in multiple blood cultures sets (Hall and Lyman, 2006). Commercial organism identification and susceptibility testing systems play increasing roles in US microbiology, and it is important that they provide accurate identification or indicate that the organism cannot be identified by their system.

*W. confusa* is not in the MicroScan data base and their PC20 and PC29 panels did not provide an identification, but the biochemical reactions pointed in the correct direction. *W. confusa* is in the data base of the RapID panel, but it provided three incorrect, high-probability identifications on the isolates from the first patient and failed to confirm the identity of the second (Table 1). 16S rDNA sequencing identified *W. confusa*.

Lee et al. (2011) reported that none of their ten *W. confusa* isolates were identified, even to the species level, by the Phoenix Automated Microbiology System (Becton Dickinson) and the Vitek 2 system (BioMerieux. Marcy l'Etoile, France). The former system identified five of the isolates as *Leuconostoc mesenteroides*, and two as *Streptococcus bovis II*, and one each was identified as *Lactobacillus lactis* and *Staphylococcus hominis.* One remained unidentified. On the Vitek, four were identified as *L. pseudo-mesenteroides*, two as *Pediococcus pentosaceus*, and one as *Steptococcus agalactiae/ equinus*. Three were not identified. We believe that it is more dangerous to provide a false characterization of the organism, than to say that the organism cannot be identified. This is expecially true in cases when the actual antibiotic susceptibilities of the organism differ from those that would be associated with the organism that was erroneously identified (*S. agalactiae*).

Matrix-assisted laser desorption ionization time-of-flight (Maldi-ToF) is increasingly being used as an organism identification system, particularly in large microbiology laboratories, because it is fast, accurate, and generally regarded as economical, despite its high initial cost. It is less costly and less labor-intensive than 16S rDNA sequencing. We sent both isolates to two reference laboratories for identification and both were identified as *W. confusa* by Maldi-ToF. Furthermore, Fusco et al. (2011) have developed a PCR assay which is specific for *W. confusa* and could be used to unambiguously identify it in foods and clinical specimens. Thus it is hoped that future *W. confusa* isolates may be rapidly and accurately identified.

VAN is generally regarded as an essential component in gram-positive and empiric antibiotic regimens. This is clearly not the case, however, and *Leuconostoc*, *Lactobacillus*, *Pediococcus*, some *Enterococcus sp*. as well as *W. confusa* have been reported to be intrinsically R to VAN (see Lee et al., 2012). Our two isolates were also R to metronidazole. They were susceptible to all other antibiotics that are generally regarded as suitable for gram-positive cocci. However, Lee et al. (2012) reported that their 10 isolates were all resistant to trimethoprim-sulfamethoxazole (MIC ≥ 128 μgm ml^−1^).

In conclusion, although *W. confusa* is a known opportunistic pathogen, it is difficult to identify in the clinical laboratory. It appears to be relatively common in fermented foods, and has been proposed as a probiotic although it is intrinsically resistant to vancomycin. It may be selected for in those who have received vancomycin therapy, as did our second patient presented above. Then it may be *identified* as a *viridans Streptococcus* because of its colony morphology, gram-stain morphology, and spot testing results, and not fully worked up or treated, as was almost the case with our first patient. Furthermore, even if it is recognized as the etiologic agent of disease, it is either not identified or mis-identified by major commercial organism identification systems that are used in clinical microbiology laboratories. Thus the incidence of disease caused by *W. confusa* is likely to be seriously underappreciated, and its inclusion in probiotic regimens should proceed with caution. Maldi-ToF is becoming more common in clinical laboratories throughout the world, and it is hoped that, if *W. confusa* is in the data base, the full spectrum of disease caused by this organism will be better recognized.

## Author contributions

All authors contributed to the acquisition and interpretation of data, the conception of the work, the drafting and revision of the manuscript and of its final approval.

### Conflict of interest statement

Dr. Salimnia has received research funding for unrelated projects from BioFire (Salt Lake City, UT, USA) and AdvanDx (Woburn, MA, USA). Dr. Lephart and Dr. Fairfax declare that the research was conducted in the absence of any commercial or financial relationships that could be construed as a potential conflict of interest.

## Author note

Some of this information was presented at the Combined Meeting of the International Congress on Antimicrobial Agents and Chemotherapy and the Infectious Diseases Society of America, Chicago, IL, USA in October 2008 (abstract number D-4004), It was also published as a case report (Salimnia et al., 2010) and as one of the cases in a review article about unusual etiologic agents of sepsis that can appear to be blood culture contaminants (Fairfax and Salimnia, 2012).
